# Engineering Novel Molecular Beacon Constructs to Study Intracellular RNA Dynamics and Localization

**DOI:** 10.1016/j.gpb.2017.04.004

**Published:** 2017-09-21

**Authors:** Zhao Ma, Xiaotian Wu, Christopher J. Krueger, Antony K. Chen

**Affiliations:** 1 Department of Biomedical Engineering, College of Engineering, Peking University, Beijing 100871, China; 2 Wallace H Coulter Department of Biomedical Engineering, Georgia Institute of Technology, Atlanta, GA 30332, USA

**Keywords:** 2′-*O*-methyl RNA, Phosphorothioate, Molecular beacon, RNA dynamics, Single-molecule RNA imaging

## Abstract

With numerous advancements in novel biochemical techniques, our knowledge of the role of RNAs in the regulation of cellular physiology and pathology has grown significantly over the past several decades. Nevertheless, detailed information regarding RNA processing, trafficking, and localization in living cells has been lacking due to technical limitations in imaging single RNA transcripts in living cells with high spatial and temporal resolution. In this review, we discuss techniques that have shown great promise for single RNA imaging, followed by highlights in our recent work in the development of **molecular beacons** (MBs), a class of nanoscale oligonucleotide-probes, for detecting individual RNA transcripts in living cells. With further refinement of MB design and development of more sophisticated fluorescence microscopy techniques, we envision that MB-based approaches could promote new discoveries of RNA functions and activities.

RNA trafficking and localization are important processes that influence cellular physiology at the epigenetic, post-transcriptional, and post-translational levels. Conventional techniques such as qRT-PCR and DNA microarrays are cell lysate-based assays that provide ensemble averages of RNA expression levels. To improve our current understanding of the role of RNAs in health and disease, there is great interest among RNA biologists to visualize individual RNAs in cells. There are currently three popular approaches — fluorescence *in situ* hybridization (FISH), the MS2 system, and molecular beacons (MBs) — commonly employed by researchers to study RNAs at the single-molecule level in various cellular contexts. Below, we discuss each technique’s usage, advantages, and limitations.

## Single-molecule RNA imaging techniques

### Single-molecule FISH

**Figure 1:**
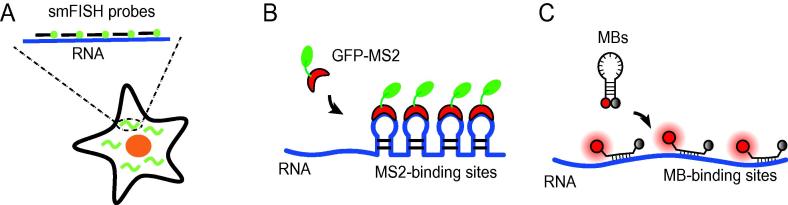
**Commonly-used techniques for single-molecule RNA imaging** **A.** smFISH labels an endogenous RNA molecule (blue line) in fixed cells using multiple oligonucleotide probes, with each probe designed to hybridize to a different region of the target RNA. **B.** The MS2 system requires engineering target RNA to harbor multiple MS2-binding sites (blue line) such that binding of GFP-MS2 fusion proteins (indicated in green and red, respectively) to the MS2-binding sites can cause the target RNA to appear as a bright fluorescent spot. **C.** MBs are stem-loop forming oligonucleotide probes that are labeled with a reporter (red circle) and a quencher (black circle). In the absence of MB target, the reporter is well-quenched. When hybridized to MB target, the fluorophore is separated from the quencher, resulting in restoration of fluorescence. When a target RNA is engineered to harbor multiple MB targets (blue line), hybridization of the MBs to MB targets can illuminate the engineered target RNA as a bright fluorescent spot. smFISH, single-molecule fluorescence in situ hybridization; MB, molecular beacon.

Single-molecule FISH (smFISH) is a powerful technique that uses multiple fluorophores to visualize specific RNA targets at the single-molecule level in cells [1]. As detection of individual fluorophores by conventional fluorescence microscopy is difficult, in order to achieve single-molecule sensitivity, multiple fluorophore-tagged oligonucleotide probes are designed to target different regions of an RNA transcript [1], [2], [3]. Hybridization of multiple probes to the same RNA molecule renders the target RNA sufficiently fluorescent upon excitation, allowing each target transcript to be imaged as a bright spot (Figure 1A, Table 1). Currently, smFISH is regarded as the gold standard approach to visualize intracellular distributions of single RNA transcripts in fixed cells and tissues [1]. However, due to the required fixation steps, RNA dynamics data cannot be easily obtained using smFISH. Additionally, smFISH requires permeabilization to allow oligonucleotide probes to enter the cell and hybridize to the target RNAs and washing to remove unbound probes. Therefore, false-negative data can sometimes result from the loss of RNAs.

**Table 1:** Feature comparison of the currently-available approaches for single-molecule RNA imaging

**Approach**	**Single molecule**	**Live-cell imaging**	***In vivo* stability**	**Imaging endogenous RNAs**	**Probe size (kDa)**	**Fluorescent selection**	**High signal-to-background**	**Refs.**
MB	Yes	Yes	Backbone chemistry dependent	Yes	∼10	Organic fluorophore	Yes	[30], [31], [32]
MS2	Yes	Yes	YES	No	∼40	Fluorescence protein	No	[4], [5], [6], [7], [8]
FISH	Yes	No	N/A	Yes	∼10	Organic fluorophore	No	[1], [2], [3]

### MS2

The MS2 system takes advantage of the ability of the bacteriophage MS2 coat protein (MCP) to bind to an aptamer sequence (known as the MS2 aptamer) with high specificity and affinity (Figure 1B, Table 1) [4], [5], [6], [7]. To enable single-RNA imaging, target RNA is genetically modified to harbor multiple tandem repeats of the MS2 aptamer. When co-expressed with an MCP-GFP fusion protein, each target RNA can then be tagged by multiple GFPs through MS2-MCP interactions, thus appearing as a bright fluorescent spot. To date, the MS2 system has been the most popular approach for imaging single engineered RNA transcripts in living cells, owing to its biostability and ease of delivery [4], [5], [6], [7], [8]. However, this approach cannot be used for imaging endogenous RNAs, and its use of fluorescent proteins limits fluorophore brightness that is necessary for high-quality imaging. Furthermore, MCP-GFP fusion proteins weigh nearly 40 kDa. Thus binding of multiple large probes to an RNA may potentially interfere with its normal activities and functions [9].

### MBs

MBs are antisense stem-loop forming oligonucleotide probes labeled with a fluorophore at one end and a quencher at the other end [10] (Figure 1C, Table 1). In the “closed” or “off” configuration, the complementary sequences flanking the loop domain anneal to form a stable stem, placing the quencher in close proximity with the reporter fluorophore, quenching its fluorescence. In the “open” or “on” configuration, target hybridization with the loop domain disrupts the stem, bringing the quencher away from the fluorophore to restore its fluorescence [10]. With careful selection of fluorophore-quencher pair, MB fluorescence can increase 20–100 fold upon hybridization to target RNA [11]. To date, MBs have been the most widely utilized tool for imaging endogenous RNA levels based on ensemble measurements [12], [13], [14], [15], [16], [17], [18], [19], [20], [21], [22], [23], [24], [25], [26], [27], [28], [29]. To achieve single-molecule sensitivity, target RNA is engineered with tandem repeats of an MB target sequence, so that multiple MBs can hybridize to a target RNA, illuminating the RNA as a bright spot [30], [31], [32]. Despite these advantages, one major limitation for the use of MBs is their biostability. The chemistry of the MB oligonucleotide backbone influences susceptibility to nuclease degradation or nonspecific protein binding, which could cause false-positive signals (FPSs) [14], [19].

### Other potential techniques for single-molecule RNA imaging

In addition to the techniques described above, other techniques, including RNA-targeting CRISPR associated protein 9 (RCas9) [33], RNA-mimics of GFP-based systems [34], and sequence-specific Pumilio-based probes [35], have been developed for visualization of subcellular localization and trafficking of specific RNA molecules based on ensemble fluorescence measurement. Further work is required to explore their potential for imaging RNA transcripts in living cells at the single-molecule level.

## MB attributes

Both the MS2 and MB systems are capable of imaging single RNA transcripts in living cells. Nonetheless, the MS2 system has been used more widely, despite the fact that MBs offer several advantages including smaller probe size, versatility in fluorophore/quencher selection, improved signal-to-background due to quenching, and the ability to image endogenous RNAs (Table 1). One major obstacle that hampers the widespread use of MBs is their tendency to be sequestered into the nucleus where they can generate FPSs as a result of nonspecific protein binding and/or nuclease degradation [14], [19], [20], [22], [31], [36].

To reduce nonspecific signals, MBs have been conjugated to macromolecules that are either too big to traverse the nuclear pores, such as quantum dots [19] and pegylated NeutrAvidins [20], or are quickly exported to the cytoplasm, such as tRNAs [37] and small interfering RNA (siRNA)-like molecules [38]. Alternatively, MBs have been synthesized with degradation-resistant oligonucleotides containing locked nucleic acids (LNA) or modified internucleotide linkages (such as phosphorothioate, PS) [20], [22], [36]. By incorporating PS linkages throughout the loop domain of a 2′-*O*-methyl (2Me) MB backbone, we have recently developed an MB architecture called the 2Me/PS_LOOP_ MB, based on the latter approach [31]. The 2Me/PS_LOOP_ MB exhibits a marginal level of FPSs in various cell types and can be used for imaging single RNA transcripts in living cells [31]. Here we highlight the work undertaken to develop 2Me/PS_LOOP_ MBs and explore their capabilities for single-molecule RNA imaging. We envision that the use of 2Me/PS_LOOP_ MBs to study RNAs in living cells can further our knowledge of the role of RNAs in health and disease.

## Optimizing MB backbone chemistry for intracellular RNA analysis

Conventional MBs, including those that are synthesized with backbones composed of DNA or 2Me RNA linked with phosphodiester bonds (DNA or 2Me MBs), can be highly sensitive to nuclease degradation. To confer nuclease resistance, a non-bridging oxygen of the phosphate may be replaced with a sulfur atom to form a chemically-modified internucleotide linkage known as the PS bond. Yeh et al. reported the first use of MBs that incorporate PS linkages throughout the probe backbone (2Me/PS_FULL_ MBs) and showed that these MBs enable detection of Coxsackie viral RNA replication for up to 12 h [20]. Supporting this finding, we showed that 2Me/PS_FULL_ MBs have longer intracellular stability and bioactivity than 2Me MBs [36]. Despite these attributes, however, we found that 2Me/PS_FULL_ MBs still cause a detectable FPS [36]. Several hours after entry, 2Me/PS_FULL_ MBs could exhibit a punctate staining pattern that can be easily misinterpreted as RNA granules or even single RNA transcripts [31]. Puncta were primarily detected in the nucleus as expected, since highly-PS-modified oligodeoxyribonucleotides (ODNs) are widely reported to bind nonspecifically to the nuclear matrix [39], [40], [41], [42], [43], [44].

**Figure 2:**
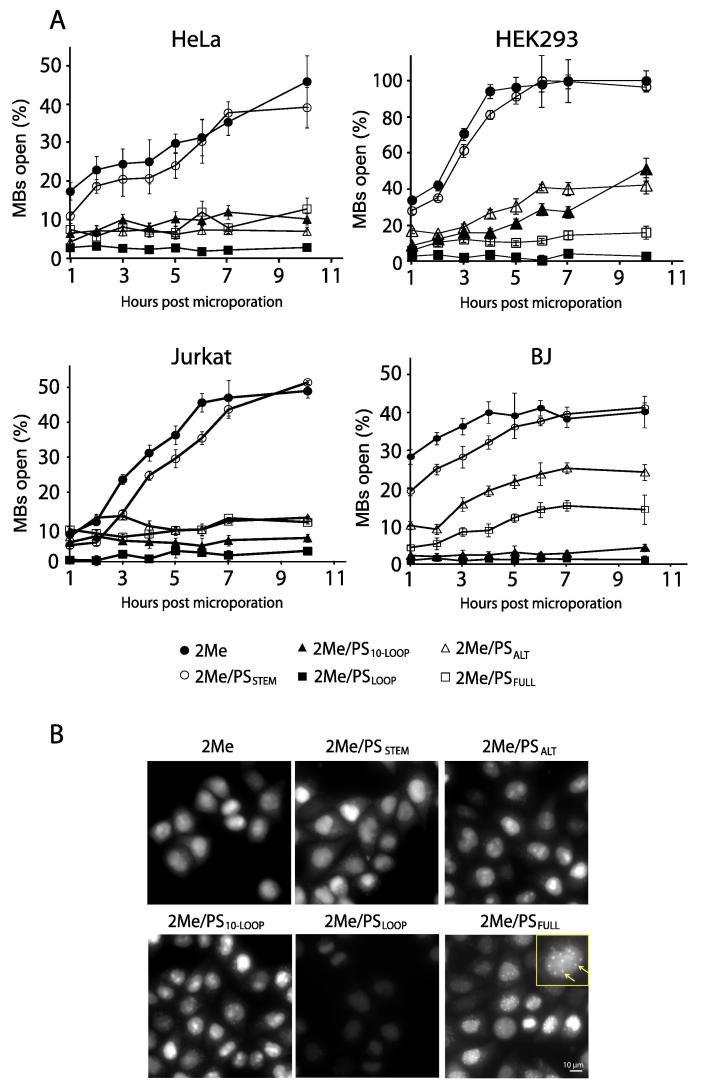
**Nonspecific opening of non-PS-modified and PS-modified MBs in living cells over time** Following microporation of 5 µM MBs that have no endogenous RNA targets into HeLa, HEK293, Jurkat, or primary BJ cells, the extent of MB opening was quantified over the course of 10 h [31]. The MBs tested include 2Me (●), 2Me/PS_STEM_ (○), 2Me/PS_10-LOOP_ (▴), 2Me/PS_ALT_ (△), 2Me/PS_LOOP_ (■), and 2Me/PS_FULL_ (□). Data are presented as mean ± S.E. from at least 30 cells. **B.** Representative images of MBs in HeLa cells, acquired at 10 h post microporation. The inset shows an expanded segment of the image. Arrows point to bright spots indicative of nonspecific binding in the nucleus (Scale bar, 10 µm). PS, phosphorothioate; MB, molecular beacon; 2Me, 2′-*O*-methyl. The graphs and images are reproduced with permission from Elsevier [31].

We hypothesized that partially-PS-modified probes may exhibit an optimal balance of nuclease resistance while avoiding excess nonspecific binding. To test this, MBs were synthesized with different numbers and distributions of PS linkages. In a variety of cell types including HEK293, HeLa, Jurkat, and primary BJ cells, we observed a general trend of MB performance relative to the degree of PS modification (Figure 2). For example, 2Me MBs and 2Me/PS_STEM_ MBs, which have a fully PS-modified stem, were both highly susceptible to nonspecific opening [31]. Incorporating PS in the loop domain significantly improved MB stability, as 2Me/PS_10-LOOP_ MBs exhibited lower FPSs than 2Me/PS_STEM_ MBs, despite having the same total number (10) of PS modifications. Consistent with this observation, 2Me/PS_LOOP_ MBs, which have a fully-PS-modified loop domain and a phosphodiester stem, exhibited even lower FPSs than 2Me/PS_10-LOOP_ MBs [31]. Only 1%−3% of the 2Me/PS_LOOP_ MBs opened nonspecifically within 10 h after delivery into several different cell types [31]. FPSs generated by 2Me/PS_LOOP_ MBs were lower than those generated by 2Me/PS_FULL_ MBs, suggesting that when the loop domain is highly PS-modified, stem domain modification is disadvantageous, as the additional PS groups can induce nonspecific binding while offering no additional increase in nuclease resistance. Overall, these findings demonstrate the feasibility of reducing the number of PS modifications in the MB backbone to reduce nonspecific binding while maintaining nuclease resistance.

Currently, debate over the primary causes of MB FPSs in living cells remains unresolved. Our findings that MBs with different PS modifications exhibit large differences in the degree of nonspecific opening can help explain why MBs open nonspecifically in cells. For example, single-stranded endonucleases appear to be the primary cause of MB nonspecific opening, as levels of FPSs are inversely correlated with the extent of PS modifications in the single-stranded loop domain [31]. Exonucleases appear to have little impact on MB degradation, as PS modifications in the stem have little effect on MB stability [31]. Presumably, the fluorophore and the quencher sterically block access by 5′- and 3′-exonucleases. The tendency of highly-PS-modified MBs to aggregate and emit FPSs is consistent with previous studies showing that PS-modified ODNs are prone to nonspecific binding to cellular proteins [39], [40], [41], [42], [43], [44]. Accordingly, the higher nonspecific signals emitted by 2Me/PS_FULL_ than 2Me/PS_LOOP_ MBs suggest that any nuclease resistance gained by PS modifications in the stem domain is offset by increased nonspecific binding due to greater number of PS modifications.

## Assessing the accuracy of 2Me/PS_LOOP_ MBs for single-molecule RNA imaging

**Figure 3:**
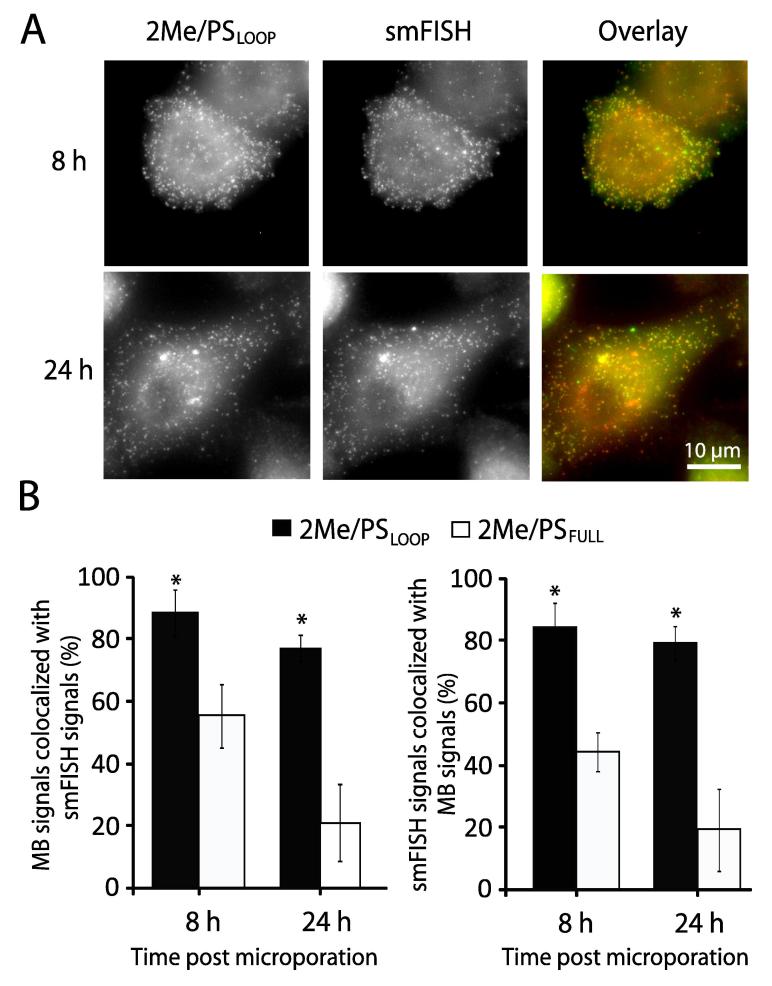
**Single-molecule RNA transcript detection using 2Me/PS_LOOP_ MBs and smFISH** HeLa cells stably expressing pEGFP-N1-32x were fixed and permeabilized 8 h or 24 h after microporation with 5 µM 2Me/PS_LOOP_ MBs. smFISH was then performed with a pool of probes designed to target EGFP to assess MB detection accuracy for single RNA transcripts. **A.** Representative maximum intensity projection images of 2Me/PS_LOOP_ MBs (Atto647NN-labeled) and EGFP smFISH probes (TAMRA-labeled) at 8 h or 24 h after microporation (Scale bar, 10 µm). **B.** The percentage of MB signals that colocalized with smFISH signals (left) and the percentage of smFISH signals that colocalized with MB signals (right) was analyzed on a cell-by-cell basis using a custom MATLAB program. Data are presented as mean ± SD from at least 10 cells. Significant difference from the 2Me/PS_FULL_ MBs is indicated with asterisks (*P* < 0.05). The images are reproduced with permission from Elsevier [31].

Our finding that 2Me/PS_LOOP_ MBs exhibit a marginal level of FPS raises the possibility of using MBs to image the dynamics and localization of single RNA transcripts in living cells with high accuracy. To determine whether 2Me/PS_LOOP_ MBs can accurately detect single RNA transcripts, we developed a plasmid construct that encodes a transcript carrying an EGFP coding sequence followed by 32 tandem repeats of an MB target sequence (pEGFP-N1-32x) [31]. As the MB target sequence and EGFP coding sequence are transcribed as one RNA molecule, we hypothesized that if MBs could hybridize to the engineered transcript, the target RNA should appear as a bright fluorescent spot reflecting MB-target hybridization. Furthermore, the MB fluorescent spot should colocalize with smFISH spots visualized using a set of probes targeting unique regions on the EGFP sequence. We found that following microporation of 2Me/PS_LOOP_ MBs and smFISH processing, bright MB and smFISH spots could be detected in the nucleus and the cytoplasm (Figure 3A). Analysis of colocalization between MB and smFISH signals in three dimensions showed nearly 90% colocalization of the MB and smFISH signals, indicating that 2Me/PS_LOOP_ MBs can detect engineered transcripts with high accuracy (Figure 3B). By contrast, in cells microporated with 2Me/PS_FULL_ MBs and processed by smFISH, only 60% of the MB signals colocalized with smFISH signals. Thus, consistent with the analysis showing that 2Me/PS_LOOP_ MBs generate lower FPS compared to 2Me/PS_FULL_ MBs, 2Me/PS_LOOP_ MBs can image single RNA transcripts more accurately than 2Me/PS_FULL_ MBs in living cells.

## Noninvasive imaging of RNA dynamics using 2Me/PS_LOOP_ MBs

**Figure 4:**
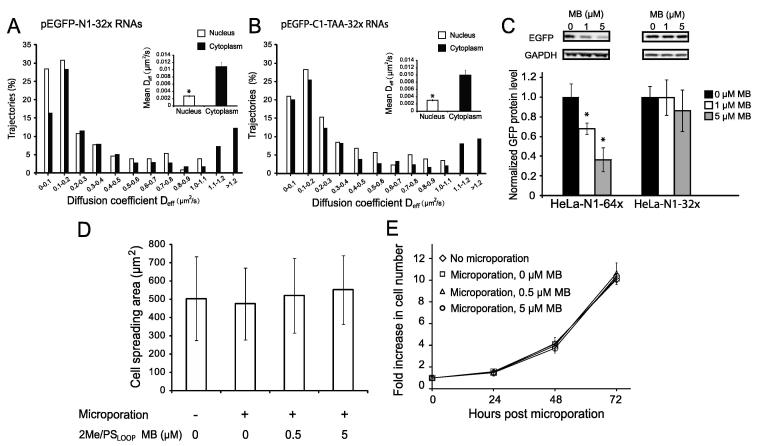
**Noninvasive imaging and measurement of RNA dynamics at the single-molecule level** Single-particle tracking analysis reveals diffusion coefficients of single pEGFP-N1-32x (**A**) or pEGFP-C1-32x (**B**) mRNAs in the nucleus and the cytoplasm of HeLa cells. Insets show average diffusion coefficients (mean ± SE) in the nucleus and the cytoplasm. Significant difference is indicated with asterisks (*P* < 0.05). (**C**) The effect of 2Me/PS_LOOP_ MBs on EGFP protein expression. Total EGFP protein level was assessed using Western blotting 24 h after microporation of 2Me/PS_LOOP_ anti-repeat MBs at concentrations of 0, 1, or 5 µM into HeLa cells stably expressing pEGFP-N1-32x or pEGFP-N1-64x RNAs. Protein expression is normalized to that in cells microporated with 0 µM MB. Total GAPDH protein level was measured as a loading control. Significant difference from 0 µM MBs is indicated with asterisks (*P* < 0.05). Average spreading (**D**) and proliferation (**E**) 24 h after microporation with different concentrations of 2Me/PS_LOOP_ MBs. All data are presented as mean ± SD of at least three independent experiments. The images are reproduced with permission from Elsevier [31].

Given their ability to detect single engineered RNA transcripts with high accuracy in live cells, 2Me/PS_LOOP_ MBs may be a promising tool to study the trafficking and localization of single RNA transcripts in real time. Figure 4A shows diffusion coefficients of single pEGFP-N1-32x RNAs in cells as measured by MB imaging. The high variance of diffusion coefficients among transcripts in both the nucleus and cytoplasm indicates a heterogeneous nature of RNA dynamics [31]. On average, RNAs in the nucleus move nearly 4 times slower than transcripts in the cytoplasm, consistent with previous findings showing that the nucleoplasm is more viscous than the cytoplasm [45]. Similar results have been obtained in cells transfected with pEGFP-C1-32x RNAs (Figure 4B), in which the 32 MB target repeats are located in the 3′-UTR. These findings suggest that an RNA transcript can be targeted by 2Me/PS_LOOP_ MBs at either 5′- or 3′-UTR. Furthermore, binding of the 2Me/PS_LOOP_ MBs to 32 tandem repeats does not cause interference with EGFP translation as seen when 64 repeats are used (Figure 4C), suggesting that MBs can be used to image target RNA containing up to 32 tandem repeats without perturbing physiological functions of target RNAs. There is no change in cell viability or cell spreading detected in cells microporated with varying concentrations of MBs (Figure 4D–E), suggesting that 2Me/PS_LOOP_ MBs do not affect cellular growth or physiology. Overall, these findings suggest that 2Me/PS_LOOP_ MBs can be a noninvasive platform for imaging single RNA transcripts in living cells.

## Conclusion

Conventional MBs have been used to image RNAs in various cellular contexts, but their propensity for nonspecific opening in living cells limits their widespread applications in studies where more sensitive detection is necessary, such as imaging RNA localization and dynamics at the single-molecule level [19], [20], [38]. We have recently developed a new MB architecture, known as the 2Me/PS_LOOP_ MB, that elicits a marginal level of FPSs in cells as compared with conventional MBs [31]. We show that 2Me/PS_LOOP_ MBs could accurately image single mRNA transcripts harboring 32 tandem repeats of an MB target sequence using conventional fluorescence microscopy. Currently, RNA dynamics at the single-molecule level has been studied primarily based on engineered RNA molecules that harbor large insertions of MB target or MS2 aptamer sites that potentially interfere with the activities of target RNAs. With further possible approaches for optimizing signal-to-background, such as fluorophore/quencher selection, and the use of more sophisticated imaging techniques, we anticipate that 2Me/PS_LOOP_ MBs can be a promising platform for live-cell, single-molecule imaging of minimally-engineered RNA molecules, or even endogenous RNA molecules, providing researchers with opportunities to study RNAs with unprecedented spatial and temporal resolutions.

## Competing interests

The authors declare no conflict of interest.
